# Fractionated Stereotactic Radiation Therapy Using Volumetric Modulated Arc Therapy in Patients with Solitary Brain Metastases

**DOI:** 10.1155/2020/6342057

**Published:** 2020-09-09

**Authors:** Chong Zhou, Youyou Xia, Pei Huang, Luan Guan, Xiaoming Shen, Dawei Hao, Meng Chen, Hongrong Ren, Lian Lian, Haitao Yin

**Affiliations:** ^1^Department of Radiotherapy, XuZhou Central Hospital Affiliated to Medical School of Southeast University, Xuzhou, Jiangsu 221009, China; ^2^Department of Radiotherapy, XuZhou Central Hospital Affiliated to Nanjing University of Chinese Medicine, Xuzhou, Jiangsu 221009, China; ^3^Department of Radiotherapy, XuZhou Clinical School of Xuzhou Medical University, Xuzhou, Jiangsu 221009, China; ^4^Department of Radiotherapy, Cancer Institute (XuZhou) of Southeast University, Xuzhou, Jiangsu 221009, China; ^5^Department of Oncology, The Affiliated Hospital of Kangda College of Nanjing Medical University (The First People's Hospital of Lianyungang), Lianyungang, Jiangsu 222002, China; ^6^Department of Oncology, Wuxi No. 2 Hospital Affiliated to Nanjing Medical University, Wuxi, Jiangsu 214002, China; ^7^Department of Oncology, Suzhou Xiangcheng People's Hospital, Suzhou 215131, China

## Abstract

**Purpose:**

To analyze retrospectively the clinical efficacy and safety for patients treated with fractionated stereotactic radiation therapy (FSRT) using volumetric modulated arc therapy.

**Methods:**

Between 2016 and 2017, 46 patients with solitary brain metastasis who underwent FSRT consisting of 25-40 Gy/5 fractions were recruited in this study. All targets within the same course received different prescriptions according to size. Toxicities were graded according to the Common Terminology Criteria for Adverse Events version 4.0.

**Results:**

The median follow-up was 11 months (3-53 months). The 6-month and 12-month local control rate calculated by Kaplan-Meier estimate was, respectively, 95% and 86%. Tumor diameter < 2.5 cm obtained 100% improved 12-month local control rate compared with 66% in those with ≥2.5 cm (*P* < 0.001). The 12-month local control calculated by Kaplan-Meier estimate was 95% in tumors with >30 Gy treatment and only 60% in tumors with ≤30 Gy treatment (*P* = 0.001). Multivariate analysis revealed that the prescription dose ≤ 30 Gy resulted in increased local failure (hazard ratio (HR), 0.14 (range, 0.019-0.95; *P* = .046)). Grade 3 or worse toxic effects were found in 5 (11%) patients, and no patient experienced surgical resection for symptomatic radioactive necrosis.

**Conclusions:**

FSRT for solid brain metastasis appears to have the advantages of a high rate of local control with a minimal risk of severe toxicity and deserves application in the clinical practice.

## 1. Introduction

Brain metastases (BMs) are a common problem in solid malignancies and the major causes of morbidity and mortality [1, 2]. Since the severity of brain metastases, a great deal of attention has been paid to obtain effective treatment of these lesions.

Evolved management was applied in the patients with brain metastases in the clinical practice. Whole brain radiation therapy (WBRT) had once been considered the standard care for BMs; however, due to the low local control (LC) rate and neurotoxicity of whole brain radiotherapy (WBRT), focal techniques with stereotactic radiosurgery (SRS) or fractionated stereotactic radiation therapy (FSRT) were considered as a better way in the treatment of the patients with 1 to 3 cm BMs [3]. The advantages of SRS include high 12-month local control rate to 70%-90% [4] and lower toxicity profiles when compared to WBRT [5]. Moreover, compared to the traditional surgery option, SRS has a greater beneficial factor to less traumatic injury and decreased risk of the leptomeningeal dissemination [6]. However, according to the report by the Radiation Therapy Oncology Group (RTOG) 90-05 study, SRS achieves a 49% of local control in BMs between 2.1 and 3 cm and of 45% in BMs between 3.1 and 4.0 cm.

Fractionated stereotactic radiation therapy (FSRT) has been used as an alternative to single-fraction SRS, with the intention to improve the therapeutic efficacy of local radiotherapy. Despite the employment of FSRT in the treatment of BMs, the optimal dose fractionation has not been established [7, 8]. In this study, we analyzed retrospectively on the efficacy and safety of FSRT using volumetric modulated arc therapy in the treatment of the patients with solitary BMs from our hospital.

## 2. Methods and Materials

### 2.1. Patients

Between January 2017 and December 2018, 46 patients with solitary BM treated with FSRT were retrospectively analyzed. Patients who underwent FSRT for untreated primary solitary cancer (small-cell lung cancer excluded)-induced brain metastases with at least 1 posttreatment magnetic resonance imaging (MRI) and a ≥70 Karnofsky performance status were included in this study. Patient and treatment characteristics are shown in Table 1. The study was approved by the institutional review board of Xuzhou Central Hospital and was carried out in accordance with ethical principles of the Helsinki Declaration.

### 2.2. Treatment

**Figure 1:**
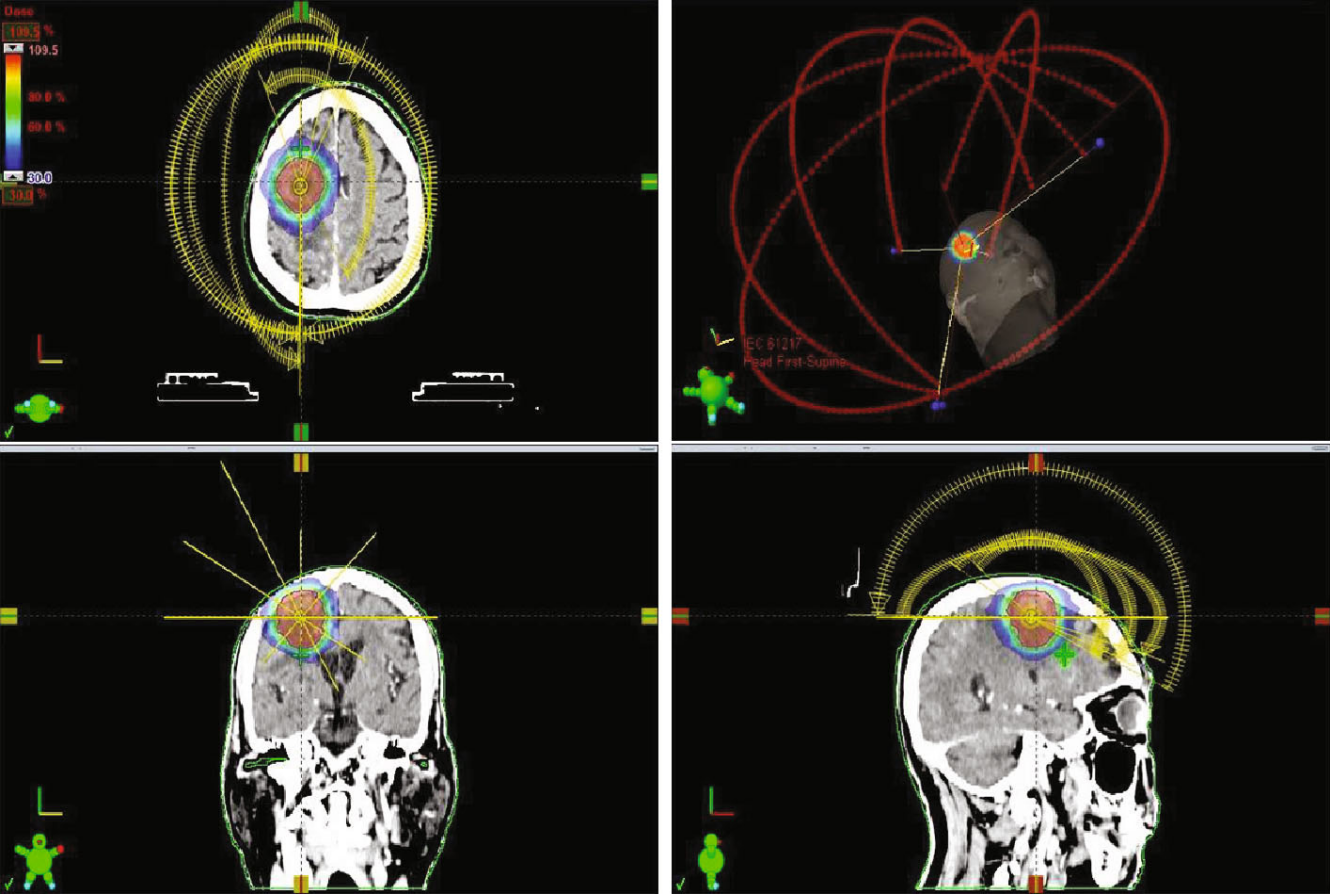
Treatment plan by volumetric modulated arc therapy (VMAT) using the 6-noncoplanar field in the case of isolated metastasis lesion in the right frontal lobe.

All cases who are scheduled for stereotactic radiotherapy were reviewed by a multidisciplinary conference. Patients were immobilized in the supine position with a stereotactic thermoplastic mask before undergoing a thin slice enhanced MRI and enhanced computed tomography (CT) scan. A slice thickness of 1.0 mm was used, and the scan range was from the top of the skull to the third cervical vertebra. MRI was performed with the simulation CT scan for improving delineation of the target and identification of the normal structure. The definition of gross tumor volume was the enhancing abnormality confirmed on the MRI and CT scan with T1 postcontrast sequence. An optional 1 to 3 mm planning target volume (PTV) expansion was employed. The total dose of prescription was 25 or 40 Gy in 5 fractions volumetrically such that the entire prescription dose was applied at least 99% of the PTV and delivered over a 7- to 14-day period. Moreover, the dosage might be adjusted by the physician according to the general condition, tumor location, and size of the individual patient. We had defined organ at risk (OAR) such as the whole brain, brain stem, optic nerve, optic chiasm, and lens. The dose constraints are the following: the brain stem to a constraint of 31 Gy, optic nerve to a constraint of 25 Gy, and minimal average dosage for whole brain and lens. All treatments were planned with the Eclipse treatment planning system (Varian Medical Systems, Palo Alto, CA). Treatment plans were prepared for the 4- and 6-noncoplanar field (as shown in Figure 1). The delivery used a 6 MV X-ray TrueBeam linear accelerator (Varian Medical Systems, Palo Alto, CA) with adjustable dose rates between 500 and 1200 MU/minute. Daily patient alignment was identified with a combination of kV orthogonal radiographs and cone beam CT for precise positioning immediately prior to treatment. For those with intracranial hypertension, mannitol and dexamethasone can be used to reduce intracranial pressure.

### 2.3. Outcome Evaluation

The clinical outcome was assessed by neurological examination, and a brain MRI was conducted 2 months post-RT and then every 2-3 months. Local progression was defined as the increase of the enhancing abnormality after the irradiated volume on serial MR imaging after the exclusion of the radiation necrosis. Distant failure was defined as the presence of new brain metastases or leptomeningeal enhancement outside the irradiated volume. Local control was evaluated in alive patients, while the overall survival analysis was carried out on all patients. Complete response (CR) was defined as the disappearance of all target lesions. Partial response (PR) was defined as a ≥30% decrease in the sum of the target lesions' diameters, and progressive disease (PD) was defined as ≥20% increase in those diameters compared to the smallest diameters from treatment. Stable disease (SD) was defined as insufficient changes to qualify as PR or PD. Non-PD (CR, PR, and SD) response was defined as locally controlled. Toxicities were classified according to the Common Terminology Criteria for Adverse Events version 4.0. Radionecrosis was evaluated and considered according to the previous description [9].

### 2.4. Statistical Analysis

Statistical analyses were performed with IBM SPSS version 23.0 (SPSS Inc., Chicago, IL). Continuous variables and categorical variables involved in the general data behavior were, respectively, expressed as the median and range and counts and percentages. The Kaplan-Meier (KM) method was employed to calculate the local control rates which were defined as the time from the date of radiotherapy to the date of progression. Univariate (UVA) analyses using the Cox proportional hazard model were carried out to identify the possible factors involved in the local failure. Statistical significance was set at *P* < 0.05 in a two-tail manner.

## 3. Results

### 3.1. Characteristics of Patients and Treatment

A total of 75 patients who underwent FSRT for solitary brain metastases between January 2017 and December 2018 were confirmed. Twenty-nine patients were excluded, including 10 patients with a previous whole brain radiotherapy history, 12 who had a nonstandard radiation dose schedule, and 7 who had no follow-up information available. Thus, a total of 46 patients were recruited in the present study. The characteristics of patients and treatment are presented in Table 1.

### 3.2. Treatment Outcome

**Figure 2:**
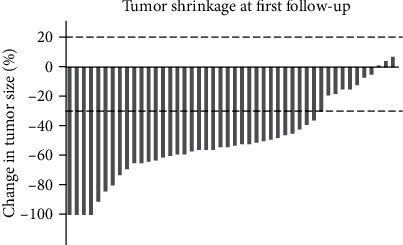
Waterfall plots showing changes in tumor size.

**Figure 3:**
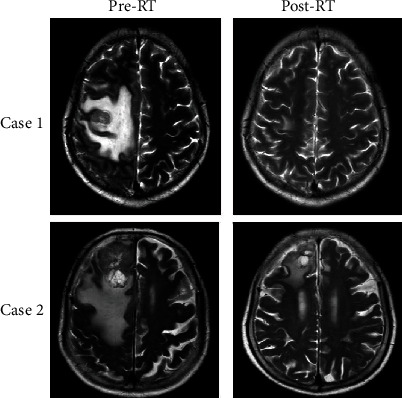
The MR images of 2 patients who underwent FSRT at pretreatment and 2 months posttreatment. Complete and partial response was found in case 1 and case 2, respectively. Case 1 is a case of lung cancer with right frontal lobe metastases which achieved complete remission in 2 months after receiving 7 Gy/F × 5F radiotherapy, whereas case 2 is a case of breast cancer with right frontal lobe metastases which achieved partial remission after 5 Gy/F × 5F radiotherapy.

The percentage changes in the tumor size at the first follow-up were shown in a waterfall plot of Figure 2. The mean tumor volume change was −30% (−100%-5%). Forty-three lesions (93.5%) and 3 lesions (6.5%) were, respectively, found in the responder group and in the nonresponder group.  Figure 3 is the representative imaging figures from 1 case of CR (case 1) and 1 case of PR (case 2) patients. The first radiological evaluation revealed a CR rate of 9%, a PR rate of 70%, an SD rate of 21%, and a PD rate of 0%.

### 3.3. Local Control Outcome

The median clinical follow-up was 11 months (ranged from 3 to 53 months). Local progression was found in 5 tumors among 5 patients at a median time of 8 months (range 5–10 months). The 6-month and 12-month local control rates calculated by Kaplan-Meier estimate were, respectively, 95% and 86%. Tumor diameter < 2.5 (*n* = 21) cm obtained 100% improved 12-month local control rate compared with 66% in those with ≥2.5 cm (*n* = 25) (*P* < .001). The 12-month local control calculated by Kaplan-Meier estimate was 95% in tumors with >30 Gy treatment and only 60% in tumors with ≤30 Gy treatment (*P* = 0.001).

### 3.4. Univariate Analyses

Table 2 shows the result of univariate analysis for local control.

### 3.5. Toxicity

A total of 46 patients in the FSRT group were evaluable for treatment toxic effects. 66 cases of different grade toxicities (irrespective of attribution) were reported by patients receiving FSRT (32 (70%) patients reported at least one toxicity). Of these, grade 3 or worse toxic effects were reported in 5 (11%) patients in the FSRT group (Table 3). No patient underwent surgical resection with symptomatic radiation necrosis. We further evaluated adverse events according to the fraction dose, and the results showed that no significant difference was found on nausea, cognitive disturbance, fatigue, and vomiting, and hearing impairment was found between the ≤30 Gy and >30 Gy groups (Table 4).

## 4. Discussion

Compared with multiple brain metastasis, the prognosis of solitary brain metastases is relatively favorable, and proper focal therapy can extend the time window for subsequent systemic therapy. In this study, we carried out a single-institution retrospective analysis to assess the efficacy and safety of FSRT in patients with solitary brain metastases. Our data showed that higher rates of local control were correlated with a smaller tumor diameter and a higher radiation dose delivery. Moreover, compared with those patients with similar characteristics treated with single-fraction SRS, well-tolerated treatment and lower CNS toxicity were found in our study [5, 10–12]. Furthermore, >30 Gy over 5 fractions was correlated with better local control compared with ≤30 Gy.

According to previous reports, the 12-month local control rates of FSRT were ranged from 52% to 95% [13–17]. Heterogeneous characteristics in previous studies including the patient population, treatment methodology, and local control definition resulted incomparable among different studies, whereas our data of the 12-month local control estimate was 86%, which seems favorable. If we set the tumor diameter to ≥3 cm larger tumors, our data of the 12-month estimate of local control totaled 66%. One important feature of our study was that we specifically excluded the tumors treated with prior focal radiation, WBRT, and surgery, unlike other studies [18, 19] with very high local control rate without these exclusions, thereby resulting in a more realistic number on the local control of FSRT.

Another specific characteristic of our study was a relatively higher ratio of smaller tumors treated with FSRT. The 12-month local control estimate for tumor diameter < 2.5 cm was 100%, which was comparable to previous studies on delivering ≥20 Gy in a single fraction [20, 21] and the FRST treatment results by Marcrom et al. The high proportion of local control observed in the present study among tumor diameter < 2.5 cm supports the growing employment of FSRT according to the literature, which confirmed small tumor size as a prediction index of improved local tumor control, and FSRT appears to be a reasonable alternative for small BMs [22, 23]. We attributed these results to the lower hypoxia ratio in small-sized BMs, and every other day, fractionated radiotherapy provides an opportunity for tumor reoxygenation [24]. Based on these exciting results, we used a similar dose and fractionation across all targets, regardless of the target size in those patients treated with FSRT in the clinical practice, which could be helpful on the improvement of the efficiency of treatment planning and delivery.

Moreover, we also observed the higher local control rate of the tumor in those patients treated with a higher dose of radiotherapy, which is consistent with the dose-dependent effects of the local tumor control in the literature on single-fraction SRS [20, 25]. Among the evaluation of the FSRT studies, conflicting reports were found on the relationship between dose and tumor control. Improved control associated with dose escalation was found in the setting of tumors receiving an equivalent dose in 2 Gy fractions (equivalent dose 2 Gy, EQD2), >35 Gy (alpha/beta = 10) or a biological effective dose using an alpha/beta = 12 (biological effective dose, BED_12_), and >40 Gy (linear quadratic cubic model) [25, 26], which was not found in other reports [11, 21]. Our study confirmed a 95% 12-month tumor control estimate with over 30 Gy in 5 fractions, which was higher than the number of 66% in those with less than 30 Gy in 5 fractions. By comparison, 30 Gy in 5 fractions equals to an EQD2 of 40 Gy (alpha/beta = 10). Therefore, the results from the current study further support the recognition that the radiation dose is dependent on the local tumor control, even with the use of multiple fractions.

Our study showed no patient with severe CNS toxicity, and grade 3 or worse toxic effects were reported in 5 (11%) patients in the FSRT group. Due to limited follow-up time of our study, it is difficult to estimate the true CNS toxicity. Increased toxicity risk could be resulted from a larger target size; however, the small event number results in failure to identify other possible contributing factors. We could not obtain the potential cutoff value of the tumor size above which 30 Gy treatment could result in increased risk of toxicity, thereby necessitating the employment of a lower dose. We did not observe increased toxicity rates among patients with over 30 Gy compared with those with less than 30 Gy, whereas other studies reported higher toxicity rates in those with higher doses [27].

The favorable toxicity profile obtained from our study could be explained by the limited follow-up and survival of our study and the late effect of radiation necrosis. The reasonable dosage and fraction regime of the radiotherapy in the majority of patients were an alternative explanation for the favorable toxicity profile in the present study since higher toxicity rates have been found in patients receiving single-fraction SRS [28]. Moreover, rotational error in treating multiple metastases with a single isocenter could result in an additional PTV margin to some targets; however, CBCT correction essentially abolished the potential rotational error observed, thereby resulting in nearly zero-margin treatments which could be reproduced consistently. In addition, prospective studies are needed to confirm the results about the lower rate of the CNS toxicity in the present study that has been shown in those with single-fraction SRS, particularly for those with larger tumors [11, 12].

There are several limitations presented in our study. Firstly, the retrospective characteristic properties of this study could result in the bias of the results obtained in our study although well-defined inclusion criteria and endpoints were employed. Secondly, the limited follow-up time in our study also could result in favorable result bias of our local control data until the obtaining of the long-term data. Third, the optimal dosing schedule remains unknown although the relatively standardized dose schedule of our institution could be helpful in analyzing the efficacy and safety of hypofractionation, which could only be confirmed after the evaluation of more patients.

## 5. Conclusion

In conclusion, we demonstrated here that in patients with solitary BM, FSRT is a safe and feasible treatment, with good brain local control and limited toxicity.

## Figures and Tables

**Table 1:** Baseline characteristics (*n* = 46).

Characteristics	Values
Age (median (IQR)), *n* (%)	57 (46.5-66)
18–59 years	25 (54)
≥60 years	21 (46)
Gender, *n* (%)	
Male	16 (35)
Female	30 (65)
Histology, *n* (%)	
Lung	26 (57)
Mammary	15 (33)
Renal cell carcinoma	2 (4)
Esophageal cancer	1 (2)
Malignant melanoma	1 (2)
Colorectal	1 (2)
Tumor diameter, *n* (%)	
<2.5 cm	21 (46)
≥2.5 cm	25 (54)
Karnofsky performance status, *n* (%)	
90-100	39 (85)
80	5 (11)
70	2 (4)
Fraction dose, *n* (%)	
5 Gy/F × 5F	7 (15)
6 Gy/F × 5F	10 (22)
7 Gy/F × 5F	21 (46)
8 Gy/F × 5F	8 (17)
PTV margin, *n* (%)	
1 mm	29 (63)
2 mm	17 (37)
Cranial nerves, *n* (%)	
Normal	46 (100)
Abnormal	0 (0)
Sensation, *n* (%)	
Normal	41 (89)
Abnormal	5 (11)
Motor, *n* (%)	
Normal	40 (87)
Abnormal	6 (13)

PTV: planning target volume.

**Table 2:** Univariate and multivariate Cox analysis of covariates related to local failure.

Variables	Univariate	*P* value	Multivariate	*P* value
	Hazard ratio (95% CI)		Hazard ratio (95% CI)	
Gender (male vs. female)	0.31 (0.034-2.75)	0.291	—	—
Age (18-59 years vs. ≥60 years)	0.76 (0.13-4.55)	0.762	—	—
Histology (NSCLC vs. breast)	0.39 (0.07-2.33)	0.301	—	—
Histology (NSCLC vs. others)	0.00 (0.00-+inf)	0.998	—	—
Tumor diameter (cm)	3.97 (1.38-11.43)	0.011	—	—
Fraction dose (Gy)	0.16 (0.047-0.54)	0.003	0.14 (0.019-0.95)	0.046
PTV margin (0 vs. 1 mm)	7.09 (0.78-64.53)	0.082	—	—

NSCLC: non-small-cell lung cancer; CI: confidence interval; PTV: planning target volume.

**Table 3:** Adverse events.

Adverse events	Grade 1	Grade 2	Grade 3	Grade 4
Nausea	5 (11%)	4 (9%)	0	0
Cognitive disturbance	5 (11%)	6 (13%)	2 (4%)	0
Fatigue	4 (9%)	2 (4%)	2 (4%)	0
Vomiting	5 (11%)	3 (7%)	0	0
Dermatitis radiation	1 (2%)	0	0	0
Hearing impairment	4 (9%)	3 (7%)	0	0
Seizure	3 (7%)	0	1 (2%)	0
Increased alanine aminotransferase	3 (7%)	1 (2%)	0	0
Anaemia	1 (2%)	0	0	0
Hyponatraemia	1 (2%)	0	0	0
Hypotension	2 (4%)	0	0	0
Meningitis	0	0	0	0
Depression	2 (4%)	0	0	0
Thrombocytopenia	0	0	0	0
Neutropenia	4 (9%)	0	0	0
Headache	2 (4%)	1 (2%)	0	0
Intracranial haemorrhage	0	0	0	0
Central nervous system necrosis	0	0	0	0

A total of 46 cases of patients were evaluated.

**Table 4:** Adverse event stratification according to the fraction dose.

Adverse events	Fraction dose ≤ 30 Gy, *n* = 17				Fraction dose > 30 Gy, *n* = 29				*P* value
	Grade 1	Grade 2	Grade 3	Grade 4	Grade 1	Grade 2	Grade 3	Grade 4	
Nausea	1	1	0	0	4	3	0	0	0.215
Cognitive disturbance	2	2	1	0	3	4	1	0	>0.999
Fatigue	1	0	1	0	3	2	1	0	>0.999
Vomiting	1	1	0	0	4	2	0	0	>0.999
Dermatitis radiation	0	0	0	0	1	0	0	0	-
Hearing impairment	1	1	0	0	3	2	0	0	>0.999
Seizure	1	0	0	0	2	0	1	0	-
Increased alanine aminotransferase	1	0	0	0	2	1	0	0	-
Anaemia	0	0	0	0	1	0	0	0	-
Hyponatraemia	0	0	0	0	1	0	0	0	-
Hypotension	1	0	0	0	1	0	0	0	-
Meningitis	0	0	0	0	0	0	0	0	-
Depression	1	0	0	0	1	0	0	0	-
Thrombocytopenia	0	0	0	0	0	0	0	0	-
Neutropenia	1	0	0	0	3	0	0	0	-
Headache	1	0	0	0	1	1	0	0	-
Intracranial haemorrhage	0	0	0	0	0	0	0	0	-
Central nervous system necrosis	0	0	0	0	0	0	0	0	-

A total of 46 cases of patients were evaluated. Nonparametric rank sum test was used for 2 group comparisons, where “-” represents noncomparable.

## Data Availability

The data used to support the findings of this study are included within the article.
